# Sleep spindle and slow wave frequency reflect motor skill performance in primary school-age children

**DOI:** 10.3389/fnhum.2014.00910

**Published:** 2014-11-11

**Authors:** Rebecca G. Astill, Giovanni Piantoni, Roy J. E. M. Raymann, Jose C. Vis, Joris E. Coppens, Matthew P. Walker, Robert Stickgold, Ysbrand D. Van Der Werf, Eus J. W. Van Someren

**Affiliations:** ^1^Department of Sleep and Cognition, Netherlands Institute for Neuroscience, Royal Netherlands Academy of Arts and SciencesAmsterdam, Netherlands; ^2^Department of Clinical Neurophysiology, Amsterdam Sleep Centre, SlotervaartziekenhuisAmsterdam, Netherlands; ^3^Department of Neurology, Massachusetts General HospitalBoston, MA, USA; ^4^Sleepvision, Berg en DalNetherlands; ^5^Department of Technology and Software Development, Netherlands Institute for Neuroscience, Royal Netherlands Academy of Arts and SciencesAmsterdam, Netherlands; ^6^Sleep and Neuroimaging Laboratory, Department of Psychology, University of CaliforniaBerkeley, CA, USA; ^7^Department of Psychiatry, Beth Israel Deaconess Medical Center and Harvard Medical SchoolBoston, MA, USA; ^8^Department of Emotion and Cognition, Netherlands Institute for Neuroscience, Royal Netherlands Academy of Arts and SciencesAmsterdam, Netherlands; ^9^Department of Anatomy and Neurosciences, VU University and Medical CenterAmsterdam, Netherlands; ^10^Departments of Integrative Neurophysiology and Medical Psychology, Center for Neurogenomics and Cognitive Research (CNCR), Neuroscience Campus Amsterdam, VU University and Medical CenterAmsterdam, Netherlands

**Keywords:** children, learning, motor skill, memory, sleep, spindles, slow waves, frequency

## Abstract

**Background and Aim**: The role of sleep in the enhancement of motor skills has been studied extensively in adults. We aimed to determine involvement of sleep and characteristics of spindles and slow waves in a motor skill in children.

**Hypothesis**: We hypothesized sleep-dependence of skill enhancement and an association of interindividual differences in skill and sleep characteristics.

**Methods**: 30 children (19 females, 10.7 ± 0.8 years of age; mean ± SD) performed finger sequence tapping tasks in a repeated-measures design spanning 4 days including 1 polysomnography (PSG) night. Initial and delayed performance were assessed over 12 h of wake; 12 h with sleep; and 24 h with wake and sleep. For the 12 h with sleep, children were assigned to one of three conditions: modulation of slow waves and spindles was attempted using acoustic perturbation, and compared to yoked and no-sound control conditions.

**Analyses**: Mixed effect regression models evaluated the association of sleep, its macrostructure and spindles and slow wave parameters with initial and delayed speed and accuracy.

**Results and Conclusions**: Children enhance their accuracy only over an interval with sleep. Unlike previously reported in adults, children enhance their speed independent of sleep, a capacity that may to be lost in adulthood. Individual differences in the dominant frequency of spindles and slow waves were predictive for performance: children performed better if they had less slow spindles, more fast spindles and faster slow waves. On the other hand, overnight enhancement of accuracy was most pronounced in children with more slow spindles and slower slow waves, i.e., the ones with an initial lower performance. Associations of spindle and slow wave characteristics with initial performance may confound interpretation of their involvement in overnight enhancement. Slower frequencies of characteristic sleep events may mark slower learning and immaturity of networks involved in motor skills.

## Introduction

The importance of sleep for learning and memory processes has been established firmly. A large number of studies in adults have shown that sleep contributes to efficient consolidation of both declarative memory—the memory for facts and events—and procedural memory—the memory for skills and procedures (Maquet, 2001; Walker and Stickgold, 2004; Stickgold and Walker, 2005; Diekelmann et al., 2009; Rasch and Born, 2013; Landmann et al., 2014). Sleep does more than merely prevent forgetting by providing a time-period without interference: for certain motor skills, sleep can even enhance performance without further training. In adults, a contribution of sleep may have been demonstrated most robustly for the consolidation and enhancement of newly learned visuomotor skills, especially of a finger-sequence tapping task (Walker et al., 2002; Morin et al., 2008; Van Der Werf et al., 2009b; Barakat et al., 2011, 2013; Albouy et al., 2013a). This task requires participants to tap a particular sequence with their fingers as fast and accurately as possible. It has been consistently shown that performance on this task saturates to a certain individual level, without further improvement unless participants try again after a period of sleep. Only if participants sleep within a certain time window after their first saturating training session, does their subsequent performance improve by about 10–20% without further training (Walker et al., 2002; Van Der Werf et al., 2009b).

What are the neuronal processes underlying this performance enhancement by sleep? Numerous studies, mostly in adults, have investigated the specific aspects of sleep-electroencephalography (EEG) that could provide clues to neuronal processes involved. These investigations have addressed qualitative aspects of the sleep-EEG macrostructure, including sleep stages, as well as quantitative aspects of the sleep-EEG, notably its power spectrum and the microstructural discrete events of sleep spindles and slow waves. Investigations of qualitative aspects of the sleep-EEG aspects of sleep revealed that overnight skill enhancement is associated with the amount of stage 2 sleep, especially in the later part of the night (Walker et al., 2002). This finding immediately points to the involvement of a specific microstructural aspect of the sleep-EEG, because stage 2 sleep is characterized by the appearance of sleep spindles (Rechtschaffen and Kales, 1968). These transient bursts of about 12–15 Hz activity reflect thalamo-cortical oscillations (Steriade, 2006). Indeed, sleep spindles have repeatedly been linked to procedural memory consolidation and enhancement (for a review see Fogel and Smith, 2011).

Along a continuum of dominant frequencies, spindles have been divided into slower and faster spindles (Feld and Born, 2012). Slow spindles dominate over frontal EEG derivations and are thought to involve the superior frontal gyrus, while fast spindles show up stronger in central and parietal EEG derivations and are thought to involve the precuneus, hippocampus, medial frontal cortex, and sensorimotor areas (Schabus et al., 2007; Dehghani et al., 2011). Relevant to the present study, the topographic representation of sleep spindles change with age (Tanguay et al., 1975; Shinomiya et al., 1999). Frontal spindles are more prominent in younger children while older children show more centroparietal spindles (Shinomiya et al., 1999).

Slow spindles are more pronounced during slow wave sleep. The slow waves of sleep represent alternating periods of hyperpolarization (down-states) and depolarization (up-states) of neurons in the cerebral cortex. Spindles are especially likely to occur at the transition to the down-state of a slow oscillation. Fast spindles occurring during slow wave sleep are more likely to occur at the transition from the down-state to the up-state (Mölle and Born, 2011). Fast spindles are most prominent during stage 2 sleep (Feld and Born, 2012). In their original study, Walker et al. (2002) showed that overnight skill enhancement is associated with the amount of stage 2 sleep, especially in the later part of the night where slow wave activity (SWA) hardly occurs. In accordance with this initial observation, fast spindles have commonly been associated with overnight enhancement of a visuomotor skill (Nishida and Walker, 2007; Tamaki et al., 2008; Barakat et al., 2011), with the overnight restoration of episodic learning ability (Mander et al., 2011) and with the overnight integration of new information in existing knowledge (Tamminen et al., 2010, 2013). Nevertheless, at least one study suggests that slow spindles rather than fast spindles are important in overnight cognitive processing (Holz et al., 2012).

In addition to spindles, slow waves have also been associated with sleep-dependent performance enhancement, possibly correlated with the role of spindles (Holz et al., 2012). The overnight enhancement of an implicit visuomotor skill is associated with the increase in slow wave power the pre-sleep training elicits in subsequent sleep (Huber et al., 2004; Määttä et al., 2010). Relevant to the present study, Kurth et al. (2012) showed in children that the maturation of simple motor skills, complex motor skills, visuomotor skills, language skills and cognitive control skills is predicted by the topographical distribution of SWA.

In contrast to adults, far less is known however about the role of sleep and associated oscillations in memory consolidation across childhood. Some studies have reported a sleep-dependent consolidation of declarative memory (Fischer et al., 2007; Backhaus et al., 2008; Wilhelm et al., 2008), but no overnight enhancement of skills (Fischer et al., 2007; Wilhelm et al., 2008). However, closer inspection of the data obtained in the finger-tapping task and mirror tracing skill tasks has indicated that children’s performance is significantly improved, both across offline periods of sleep and wakefulness (Wilhelm et al., 2008; Prehn-Kristensen et al., 2009). Moreover, 9- and 12-year old children showed less susceptibility to daytime interference of a newly acquired motor memory than 17 year olds (Dorfberger et al., 2007). This supports the interpretation that children have the capacity for memory consolidation over periods of both sleep and wakefulness, the latter being diminished or even lost with the development into adulthood.

With respect to the involvement of sleep specific sleep oscillations in performance enhancement in children, Kurdziel et al. (2013) found that a daytime nap in 4 year old children enhanced recall on a hippocampal-dependent visuospatial task resembling the card-deck “Memory” game. Moreover, sleep spindle density during the intervening nap was positively correlated with the memory performance benefit (*r* = 0.65). However, these memory associations may have been secondary to a negative correlation of spindle density with initial baseline memory performance (*r* = −0.67), thereby offering more improvement opportunity in children with lower baseline ability. Of note, a negative correlation of spindle density with baseline performance was also reported in 4–8 year old children (Chatburn et al., 2013).

Building on these prior findings, the first aim of the present study was to address the hypothesis that motor skill enhancement is dependent on sleep in school-aged children, as it has been reported to be in adults. The second aim was to determine whether both baseline motor skill performance and offline enhancements were significantly predicted by specific aspects of the sleep-EEG. In particular, we focused on the role of fast and slow sleep spindles and slow waves of sleep. Thirdly, to attain support for the hypotheses beyond observational correlations between sleep and memory in children, we implemented an experimental manipulation aimed at changing spindles and slow waves, thus exploring causality. Pharmacological manipulation of spindle density affects sleep-dependent performance enhancement of sequence finger tapping (Rasch et al., 2009) but may not easily be approved of by medical ethics committees for application in children, and may induce other systematic effects. We therefore aimed to manipulate spindles and slow waves only during slow wave sleep, using a validated selective acoustic interference of sleep at the first occurrence of slow waves (Van Der Werf et al., 2009a). This method selectively and effectively suppresses slow waves (Van Der Werf et al., 2009a) and therefore their co-occurrence with spindles, allowing for a better discrimination of the role of sleep spindles vs. slow waves, and sleep spindles that occur in stage 2 vs. those that occur in slow wave sleep. Moreover, since fast spindles are more prominent during stage 2 sleep and slow spindles occur more pronounced during slow wave sleep, selective suppression of slow waves further offers the ability to more clearly disambiguate the role of fast vs. slow spindles in memory processing.

## Methods

### Participants

Participants were recruited through a national competition designed to promote an interest in science amongst primary schools in the Netherlands. The two final school classes of the winning school were invited to take part in the current study. For ethical reasons, all children for which informed consent was obtained participated in the experiment, including children with diagnosed psychiatric or neurological illnesses. By allowing them to participate, their condition remained concealed to their peers. Their data were however excluded from analysis. The data of two participants were excluded because of a diagnosis with Pervasive Developmental Disorder—Not Otherwise Specified (PDD-NOS). Useful data were obtained from 30 participants, 19 females (10.7 ± 0.8 years; mean ± SD). No apparent sleep disorders were present as indicated by Dutch translations of the abbreviated Child’s Sleep Habits Questionnaire (CSHQ, cutoff score 41; Owens et al., 2000b) and Sleep Disturbance Scale for Children (SDSC, cutoff score 39; Bruni et al., 1996) filled out by the parents and the Sleep Self Report (Owens et al., 2000a) filled out by the children. The local medical ethics committee approved of the procedures and written informed consent was obtained from the parents.

### Procedural task

The current study used a paradigm frequently employed to examine sleep-dependent procedural performance enhancement in adults: the finger-tapping task (Karni et al., 1995; Walker et al., 2002). The task consists of two sessions: an initial learning acquisition session, followed by an offline time period of either wake or sleep, after which there was a delayed recall test session to investigate the development of offline performance changes, relative to the end of the initial acquisition session. In the current version, each learning session consisted of 12 trials of 23-s duration, separated by 20-s breaks. The delayed recall session consisted of six additional trials, again separated by 20-s breaks. During a trial, participants were asked to continuously tap a five-digit sequence on a computer keyboard (e.g., 4-1-3-2-4) as fast and as accurately as possible with their non-dominant hand. Four parallel versions of the task were used and these were counterbalanced across participants and across the four experimental conditions: 41324, 32413, 14231 and 23142.

Key-presses were recorded using E-prime (Psychology Software Tools Inc., Pittsburgh, USA) and processed to derive two main variables of interest for each trial: (1) speed, i.e., the number of correct sequences per 23-s trial; and (2) accuracy, i.e., the percentage of key taps that resulted in correct sequences, relative to all key taps.

### Experimental design

**Figure 1:**
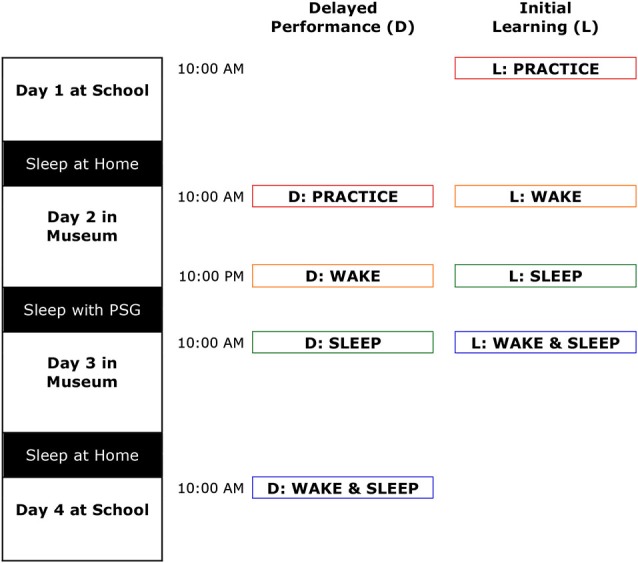
**The study spanned four consecutive days**. Children performed the motor skill task at school on day 1 and 4, and in the Science Museum on day 2 and 3. The first and last nights were regular non-monitored nights during which the children slept at home. Children underwent PSG and slept in the Science Museum during the second night. After practicing initial learning and delayed performance (black) the task was performed across three intervals: 12 h of wake (red), 12 h containing sleep (blue) and 24 h including wake and sleep (purple). Learning (L) consisted of 12 trials of 23 s duration; delayed (D) of six more trials.

Using a repeated-measures design, participants performed finger-tapping learning and recall sessions three times, preceded by an additional initial acquisition learning (L) and recall (R) practice sessions to get familiar with the task. Assessments spanned four consecutive weekdays with morning sessions at 10:00 AM and the evening session at 10:00 PM. As indicated in Figure 1, after the initial learning and recall practice sessions, performance changes were assessed in a fixed order over the following intervals: (1) 12 h containing wake (the *Wake* interval); (2) 12 h including sleep (the *Sleep* interval); and (3) 24 h including both wake and sleep (the *Wake & Sleep* interval). In the 12-h *Sleep* interval, participants stayed in individual bedrooms in a purposefully built sleep-lab in the Science Museum “Nemo” (Amsterdam, Netherlands) for polysomnography (PSG) recordings. Every three children were supervised by at least one sleep technician. The nights in-between the learning and recall training sessions and the *Wake & Sleep* interval were spent at home, during which the children slept in their own bed as per usual.

### Polysomnography (PSG)

During the 12-h *Sleep* interval, participants were fit with eight Au electrodes: two for electroencephalography (EEG) on frontopolar (FPz) and central (Cz) positions according to the 10–20 system, two for electrooculography (EOG) placed diagonally across the eyes, two for electromyography (EMG) attached submentally, a ground electrode positioned on the forehead and a reference electrode (A1) fit on the left mastoid. Polysomnography was performed using the Embla A10 system (Flaga hf, Reykjavik, Iceland). Data were recorded online, and transferred onto a personal computer. The Embla A10 system initially samples the data at 2000 Hz and subsequently down-samples it digitally to 200 Hz. Filtering was limited to the Embla’s integrated highpass DC filter at 1 Hz (−3 dB at 0.3 Hz) and 50 Hz notch filter (1 Hz bandwidth).

During the night that the children spent in the sleep-lab, they were randomly assigned to one of three acoustic manipulation conditions. All children wore in-ear headphones. The first condition has been described previously (Van Der Werf et al., 2009a) and aimed at suppressing slow wave sleep. In brief, we developed a custom analysis plug-in for the Somnologica 2 software (Flaga, Reykjavik, Iceland) that performed online calculation of the relative contribution of the SWA band (0.4–4 Hz) to the frequency spectrum as a measure of the depth of sleep. When the contribution of SWA exceeded a threshold level, the headphone emitted a beeping noise that continued to increase in amplitude in six discrete steps until it reached a maximum. The sound continued until the level of SWA dropped below the threshold. To avoid erroneous inclusion of slow EOG signals in the 0.4–4 Hz EEG band, the sound was not emitted when the signals from the two EOG leads were negatively correlated, reflecting conjugated eye movements; a positive correlation reflects leakage of SWA into the EOG leads. Using this system, we have successfully achieved slow wave sleep suppression in elderly volunteers (Van Der Werf et al., 2009a).

The second acoustic manipulation condition concerned a yoked control group, who received the same auditory stimuli, but unrelated to their own slow wave sleep. They received a copy of the auditory stimuli that were given in a closed-loop way to their sleeping neighbor. Finally, the third, placebo, condition consisted of merely wearing the in-ear headphones without providing any acoustic stimulation.

Children were blinded to the condition they were assigned to and were told that tones would be played in the night, but that they might not become aware of them.

### EEG analysis

#### Macrosleep

Electroencephalography was scored visually, blinded to the condition, in 30-s epochs using Somnologica software (Flaga hf, Reykjavik, Iceland) according to standard sleep scoring criteria (Rechtschaffen and Kales, 1968) with the adaptation of viewing EEG at 100 μV/cm instead of the recommended 50 μV/cm, to account for the very large amplitude of sleep EEG oscillations in children (Piantoni et al., 2013a). Macrosleep variables quantified were Time In Bed (TIB), Total Sleep Time (TST), Sleep Onset Latency, Latency to the First REM epoch, Wake after Sleep Onset, Sleep Efficiency and the Percentages of Stage 1, 2, SWS and REM sleep relative to TST.

#### Preprocessing for quantitative EEG analysis

The visual scoring included a rating of presence of artifacts. Epochs of 30 s that contained even the slightest artifact, including an arousal, were omitted from quantitative EEG analyses.

#### Spindles

Automated spindle detection was performed using a previously reported algorithm (Ferrarelli et al., 2007) implemented in Matlab (The MathWorks Inc, Natrick, USA). Artifact-free EEG in stages S2, S3, and S4 across the entire night was bandpass-filtered between 9 and 15 Hz using an infinite impulse response filter (Figures 4A,B). We then computed the time-course of the amplitude by taking the envelope of the filtered signal (Figure 4B). For each channel and participant, the mean of the envelope over the artifact-free stages S2, S3, and S4 was used to calculate the upper threshold: all amplitude fluctuations of the filtered signal surpassing 4.5-fold the average amplitude value calculated above were considered putative spindles (Figure 4C). The beginning and end of each spindle was defined by a lower threshold, set at 25% of the upper threshold value (Figure 4C). A minimal duration of 450 ms was used to avoid the detection of brief events. Visual inspection of the performance of the automated algorithm indicated the need of slight adaptations in the parameter settings as compared to the settings used in Ferrarelli et al. (2007), in particular we used a lower threshold for spindle detection and we applied an additional smoothing window. Spindle outcome variables were: duration, maximal amplitude, duration × maximal amplitude, and density (the number of spindles per valid epoch of sleep) of slow (frequency <12 Hz) and fast (frequency ≥12 Hz) spindles.

**Figure 2:**
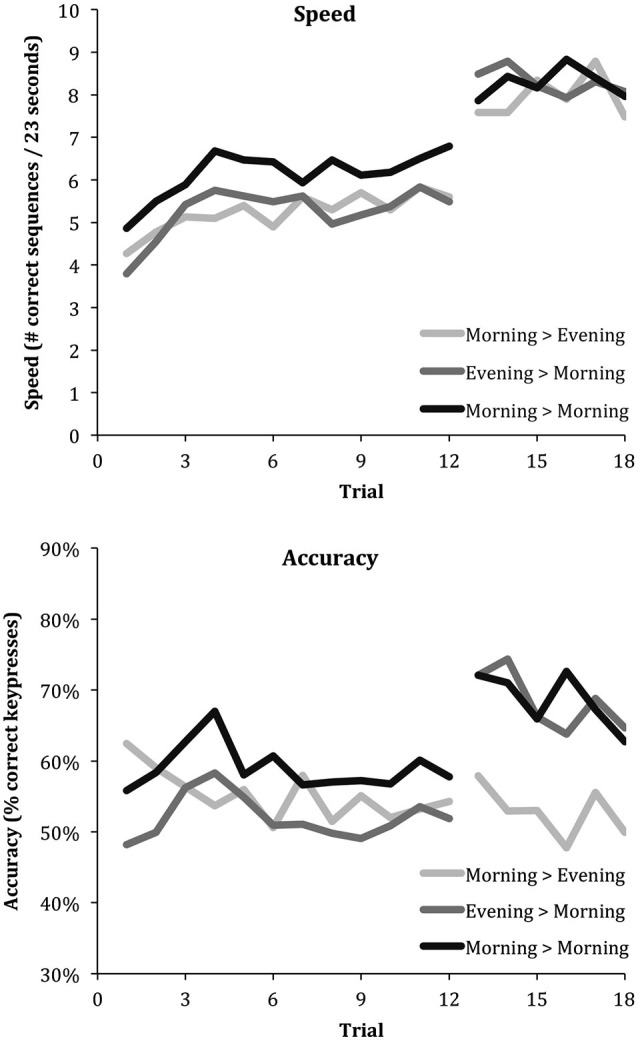
**Learning curves for speed and accuracy across the three intervals (Morning > Evening, Evening > Morning and Morning > Morning)**. Irrespective of sleep, all intervals show an increase in speed following a period without training. On the other hand, only the intervals containing sleep induce an increase in accuracy.

**Figure 3:**
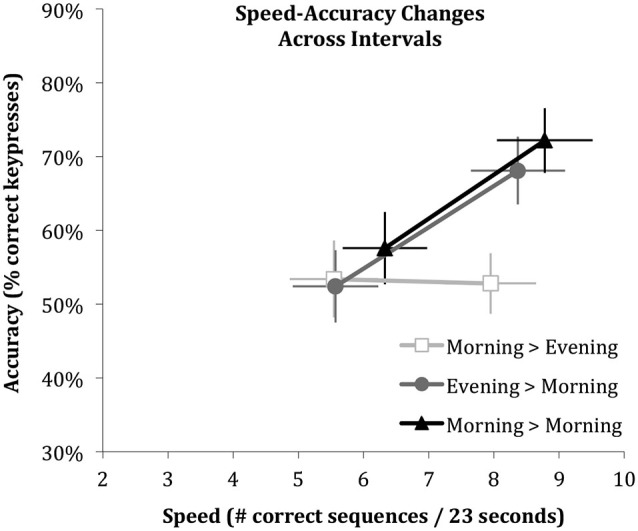
**Performance changes across the three intervals shown as vectors of speed and accuracy**. Error bars indicate standard errors of the mean derived in mixed effect analyses. Note that speed increases across all intervals, whereas accuracy improves only across the intervals that include sleep.

**Figure 4:**
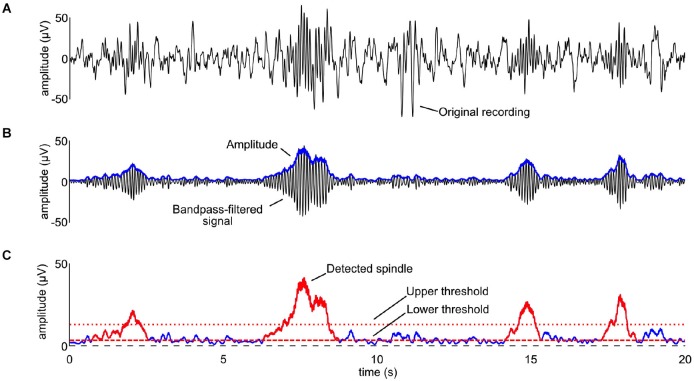
**The spindle detection procedure described in detail. (A)** The original recording for one participant in stage S2. **(B)** The signal was bandpass-filtered between 9 and 15 Hz (black line) and the time-course of its amplitude was computed by rectifying the signal, applying a low-pass filter at 4 Hz (Nir et al., 2007), and multiplying by √2 (blue line). **(C)** An upper threshold equal to the 4.5 times the mean of the amplitude in stages S2, S3, and S4 was used for the detection of the spindles (dotted red line). A lower threshold was used to define the beginning and end of each spindle (dotted dashed line). Detected spindles are shown as red traces superimposed on the time-course of the amplitude. Note that the *x*-axis is the same for all the panels, while the *y*-axis in the bottom panel is twice as large as that of panels **(A)** and **(B)**.

#### Slow waves

Automated slow wave detection was performed using an algorithm based on previously published methods (Massimini et al., 2004; Riedner et al., 2007) implemented in Matlab (The MathWorks Inc, Natick, USA). Artifact-free EEG classified as S2, S3 and S4 was high-pass filtered at 0.16 Hz (transition band width = 0.02 Hz) and low-pass filtered at 4 Hz (transition band width = 0.6 Hz), using a least-square zero phase-shift 200th order FIR filter. In the filtered signal, slow waves were defined by the appearance of a particular order of occurrences: a downgoing zero crossing, a negative peak, an upgoing zero crossing, a positive peak, and a final downgoing zero crossing. A slow wave was counted if the duration between the downgoing and upgoing zero crossing (the negative half wave) was between 0.3 and 1 s. No amplitude criteria were set. Slow wave outcome variables were the durations and peak amplitudes of the negative and positive half-wave and total wave (using downward and upward zero-crossings, see e.g., Heib et al., 2013); the steepness of the rising slope of the negative half-wave (see Piantoni et al., 2013b); and the density (the number of slow waves per epoch of NREM stage 2 and SWS sleep; see Piantoni et al., 2013b).

### Statistical analysis

The four paragraphs below describe the analysis plan, respectively addressing: the effect of sleep on performance; the association of sleep variables with performance baseline and overnight enhancement; the effect of acoustic perturbation on sleep outcome variables; and the effect of acoustic perturbation on performance outcome variables.

#### Effect of sleep on performance

In order to maximally exploit the variance information of speed and accuracy data of individual trials, they were not averaged, but rather analyzed using mixed models (MLwiN, Centre for Multilevel Modeling, Institute of Education, London, UK). Mixed models take an interdependence of data points into account; allowing trials to be nested within sessions, which are subsequently nested within participants. Maximal use of information was attained by including trials at the level of performance saturation (see Figure 2: the last six trials of the learning sessions and all six trials of the recall sessions).

In order to evaluate the effect of sleep on initial (baseline) performance and performance enhancement, the dependent variables “speed” and “accuracy” assessed over all sessions were analyzed using the regression equation:

$${\text{Y}}_{\text{ijkl}}{\text{= ß}}_{\text{0ijkl}}{\text{+ ß}}_{\text{1}}{\text{* Recall}}_{\text{jkl}}{\text{+ ß}}_{\text{2}}{\text{* Slept}}_{\text{jkl}}{\text{+ ß}}_{\text{3}}{\text{* Recall * Slept}}_{\text{jkl}}$$

where: *Y* is the dependent variable (either “speed” or “accuracy”), measured on trial i of the initial learning vs. delayed part j of session k of child l; ß_0_ is the model intercept; “Recall” is a binary (dummy) variable that indicates whether the trial was a recall (1) or initial learning (0) trial; “Slept” is a binary (dummy) variable that indicates whether the present session was (1) or was not (0) preceded by a previous session followed by a period of sleep; “Recall*Slept” is a binary (dummy) variable that indicates the interaction between “Recall” and “Slept”. This interaction represents the sleep-dependent effect on recall. The variable is 1 for recall trials in sessions that are separated from the previous session by a period including sleep and 0 for all learning trials and recall trials in sessions that are separated from the previous session by a period of wakefulness only.

### Association of sleep variables with performance baseline and overnight enhancement

In order to evaluate the effect of sleep variables assessed during the third night on baseline performance and performance enhancement across that night, the dependent variables “speed” and “accuracy” were analyzed using the regression equation:

$${\text{Y}}_{\text{ijkl}}{\text{ = ß}}_{\text{0ijk}}{\text{+ ß}}_{\text{1}}{\text{* Recall}}_{\text{jk}}{\text{+ ß}}_{\text{2}}{\text{* Sleepvariable}}_{\text{jk}}{\text{+ ß}}_{\text{3}}{\text{* Recall * Sleepvariable}}_{\text{jk}}$$

where: *Y* is the dependent variable (either “speed” or “accuracy”), measured on trial i of the initial learning vs. delayed part j of child k; ß_0_ is the model intercept; “Delayed” is a binary (dummy) variable that indicates whether the trial was a delayed (1) or initial learning (0) trial; “Sleepvariable” is the sleep variable of interest in the current analysis and indicates the nonspecific (i.e., sleep-unspecific) association of the sleep variable with performance; “Delayed*Sleepvariable” represents the interaction between “Delayed” and “Sleepvariable”. This interaction represents the sleep variable-dependent change in performance from the initial learning session to the delayed session.

#### Effect of acoustic perturbation on sleep outcome variables

Kruskal-Wallis tests (SPSS 12.0.1 for Windows, Chicago, USA) were applied to evaluate differences in macrosleep and quantitative EEG variables between acoustic perturbation conditions. The more robust Kruskal-Wallis tests were preferred over ANOVAs because variance estimates, although not precise due to the small and unequal sample sizes of the three groups, seemed to differ for some variables.

#### Effect of acoustic perturbation on performance outcome variables

In order to evaluate the effect of sleep perturbation, during the third night, on baseline performance and performance enhancement across that night, the dependent variables “speed” and “accuracy” were analyzed using the regression equation:

$${\text{Y}}_{\text{ijkl}}{\text{ = ß}}_{\text{0ijk}}{\text{+ ß}}_{\text{1}}{\text{* Delayed}}_{\text{jk}}{\text{+ ß}}_{\text{2}}{\text{* Slow Wave Triggered Sound}}_{\text{jk}}{\text{+ ß}}_{\text{3}}{\text{* YokedSound}}_{\text{jk}}{\text{+ ß}}_{\text{4}}{\text{* Delayed * Slow Wave Triggered Sound}}_{\text{jk}}{\text{+ ß}}_{\text{5}}{\text{* Delayed * YokedSound}}_{\text{jk}}$$

where: *Y* is the dependent variable (either “speed” or “accuracy”), measured on trial i of the initial learning vs. delayed part j of child k; ß_0_ is the model intercept; “Delayed” is a binary (dummy) variable that indicates whether the trial was a delayed (1) or initial learning (0) trial; “SlowWaveTriggeredSound” and “YokedSound” are two dummy binary (dummy) variables that code whether (1) or not (0) the child was assigned to the stimulation condition; both are zero for the control condition; “Delayed*SlowWaveTriggeredSound” and “Delayed*YokedSound” represent the interactions of “Delayed” with the conditions. These interactions represent the condition-dependent change in performance from the initial learning session to the delayed session.

For all mixed effect models, the significance of the regression coefficient estimates of interest was evaluated using the Wald test, that calculates a *z*-value as the ratio of the coefficient estimate over its standard error (Twisk, 2003). Effects with *P* < 0.05 were regarded significant.

## Results

In three children, one of the learning sessions was missed, twice because of equipment malfunctioning, once because the subject did not feel well temporarily. The corresponding delayed trials were omitted accordingly. In two participants one consistently noisy sleep-EEG channel (Cz) was omitted from analyses. Completely artifact-free data used for quantitative EEG analysis accounted for 65.1% (±1.3%; SEM) of the total PSG data acquired. The percentage of epochs containing even the slightest artifact slowly increased during the sleep period from 23% in the first hour of the night to 43% in the last hour of the night.

### Effect of acoustic perturbation on sleep and performance outcome variables

Counter to the impact in adults (Van Der Werf et al., 2009a), Kruskal-Wallis tests on acoustic perturbation confirmed no significant differences in either macrosleep outcome variables or NREM oscillations of the sleep recordings of children included in the closed loop slow wave suppression group (*n* = 9), the yoked control group (*n* = 10) and the no-noise group (*n* = 11): TIB (*P* = 0.759), TST (*P* = 0.847), Sleep Onset Latency (*P* = 0.758), Latency to the First REM epoch (*P* = 0.458), Sleep Efficiency (*P* = 0.742) and the percentages of Wakefulness (*P* = 0.192) Stage 1 (*P* = 0.599), 2 (*P* = 0.659), SWS (*P* = 0.493) and REM sleep (0.373), spindle variables (FPz: 0.194 < all *P* < 0.706, Cz: 0.257 < all *P* < 0.913) or slow wave outcome variables (FPz: 0.135 < all *P* < 0.966, Cz: 0.662 < all *P* < 0.981). The analyses confirm that children slept through the acoustic perturbation without any measurable effect on their macrosleep or quantitative sleep variables. Mixed effect models confirmed that the overnight change in motor skill speed and accuracy were not affected by either the Slow Wave-Triggered or Yoked Sound (0.505 < all *P* < 0.975). Due to the lack of effect of acoustic stimulation, further results aggregate the data of all children, irrespective of condition.

### Effect of sleep on performance

Figure 2 shows the trial-by-trial average speed and accuracy for the *Wake*, *Sleep* and *Wake & Sleep* conditions. Mixed effect models evaluated how speed and accuracy were affected at delay (“Delayed” effect), by sleep between the present and previous session (“Slept” effect), and by a sleep-dependent effect specific to delay (“Slept”*“Delayed” interaction), i.e., showing only in the previously trained sequences but not in the subsequent newly trained sequences. According to the output generated by mixed effect model estimation, all estimated effects are shown as average ± standard error of the mean.

The analysis showed a very significant “Delayed” effect on speed, which increased on average from the six final training trials to the six delayed trials by 2.617 ± 0.421 correct sequences (48% of the initial performance that was 5.459, *Z* = 6.216, *P* = 5E^−10^). Overall speed, i.e., aggregated over both delayed trials and initial learning trials, did not depend on whether children had slept in between the present and prior session (“Slept” effect: 0.339 ± 0.338 correct sequences, *Z* = 0.947, *P* = 0.34). There was no “Delayed*Slept” effect on speed, indicating that the performance increase occurred independently of whether children had slept in between the initial learning and delayed session; neither was there a sleep-dependent delay-specific effect on speed (−0.096 ± 0.570, *Z* = −0.168, *P* = 0.87). Thus, children showed strong speed improvements both after a period of sleep and after a period of wakefulness, selectively for the previously learned sequences, without affecting performance on the subsequent newly trained sequences.

In contrast, there was a highly significant sleep-dependent effect on accuracy, which increased by 12.4 ± 4.6% (26% of the initial accuracy that was 47.6%, *Z* = 2.696, *P* = 0.007) specifically for the delayed trials, without any sleep-unspecific delayed effect (−1.6 ± 3.5%, *Z* = −0.457, *P* = 0.65) or non-delay-specific effect of sleep (−3.0 ± 4.6%, *Z* = −0.652, *P* = 0.51). Thus, children showed a strong reduction in error rates only after a period of sleep and only for the previously learned sequences, without affecting performance on the subsequent newly trained sequences, meaning that sleep did not affect performance on subsequent newly trained sequences.

Figure 3 shows an integrated view of the changes in speed and accuracy from initial learning to retesting of the same sequence for each of the three intervals (*Sleep, Wake, Wake & Sleep*) as vectors. It illustrates how speed increases independent of whether or not the interval contained sleep (rightward change), while accuracy increases only if the interval contained sleep (upward change).

### Association of macrosleep variables with performance baseline and overnight enhancement

The overnight increase in accuracy was more pronounced in children with a higher percentage of SWS (0.85 ± 0.43% per % more SWS, *Z* = 1.977, *P* < 0.05). Given that the range of SWS percentages found in the group of children was 23% to 46%, this finding suggests that the increase in accuracy may differ up to 20% (0.85*23%: for every % more SWS a child shows, it has a 0.85% higher accuracy, and there is a difference of 23% between the child with the lowest and highest percentage slow wave sleep).

### Spindle characteristics and their association with performance baseline and overnight enhancement

**Figure 5:**
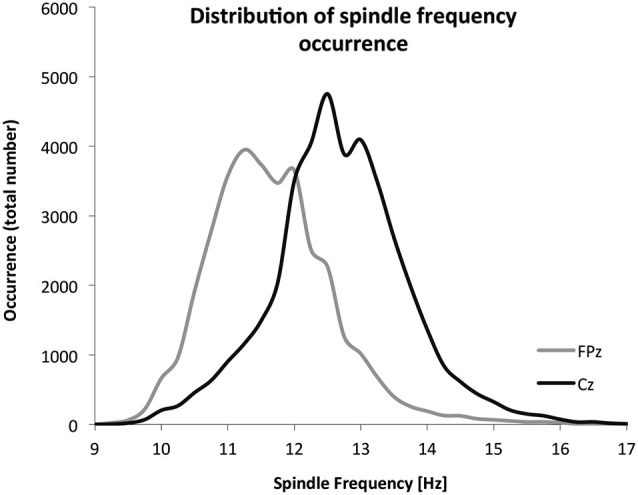
**The frequency distribution of all detected spindles**. A total of 37,177 spindles were detected on FPz (30 children), and a total of 39,951 spindles were detected on Cz (28 children).

Given the frequency distribution of spindles at FPz and Cz (Figure 5), the cut-off to discriminate fast and slow spindles was set at 12 Hz. Spindles were more prevalent and of a faster frequency at Cz. Table 1 summarizes the spindle characteristics. Mixed effect models evaluated the association of spindle characteristics with both the overall level and the overnight change in performance. Significant effects were found only for the density of slow and fast spindles.

**Table 1:** **Sleep variables averaged over all children**.

	Mean ± SEM	Mean ± SEM
**Macrostructure characteristics**
Time in Bed (TIB) (min)	464.7 ± 1.76
Total Sleep Time (TST) (min)	432.0 ± 4.62
Sleep Onset Latency (min)	19.3 ± 2.34
First REM Latency (min)	94.3 ± 6.92
Wake After Sleep Onset (min)	9.4 ± 2.10
Sleep Efficiency %	92.9 ± 0.88
% Stage 1 (of TST)	3.4 ± 0.63
% Stage 2 (of TST)	39.0 ± 1.32
% Slow Wave Sleep (of TST)	34.0 ± 1.06
% REM (of TST)	23.7 ± 0.82
**Sleep spindle characteristics**	**FPz**	**Cz**
Duration (ms)	1100.96 ± 24.70	1206.72 ± 17.99
Amplitude (μV)	17.84 ± 0.66	27.74 ± 0.80
Duration*Amplitude (μVs)	20.37 ± 1.00	34.52 ± 1.29
Frequency (Hz)	11.55 ± 0.06	12.49 ± 0.08
Density (# / 30 s epoch)	2.24 ± 0.07	2.48 ± 0.06
**Slow wave characteristics**
Duration negative half wave (ms)	457.44 ± 1.32	451.52 ± 1.35
Duration positive half wave (ms)	333.36 ± 1.66	327.56 ± 1.69
Total duration (ms)	790.80 ± 2.76	780.14 ± 2.98
Amplitude negative half wave (μV)	−55.63 ± 1.92	−76.87 ± 2.42
Amplitude positive half wave (μV)	58.62 ± 2.36	76.00 ± 2.59
Peak-to-peak amplitude (μV)	126.96 ± 4.53	162.33 ± 5.48
Up-slope negative half wave (μV/ms)	0.31 ± 0.01	0.43 ± 0.01
Density (# / 30 s epoch)	23.72 ± 0.19	22.59 ± 0.19

With respect to overall performance, i.e., not specific for overnight enhancement and including all trials, children with a higher density of slow spindles at either Cz or FPz have lower overall speed (−5.45 ± 1.63 correct sequences/spindle per sleep epoch, Z = −3.342, *P* < 0.001) and accuracy (−27.5 ± 12.4%/spindle per epoch, Z = −2.218, *P* < 0.03). In contrast, children with a higher density of fast spindles have a higher overall speed (4.46 ± 1.52 correct sequences/spindle per sleep epoch, *Z* = 2.919, *P* < 0.004) and, if anything, a non-significant higher accuracy (15 ± 11.5% per spindle per epoch more, *Z* = 1.304, *P* = 0.19).

With respect to the overnight enhancement of performance, children with a higher density of slow spindles show a stronger overnight increase in accuracy (16.1 ± 6.8% more increase/spindle per epoch, *Z* = 2.368, *P* = 0.02), but not speed (*P* = 0.45). In contrast, individual differences in fast spindle density did not show an association with overnight change in either speed (*P* = 0.61) or accuracy (*p* = 0.39).

Because slow spindles occurred more frequently at FPz and fast spindles more at Cz, we performed ancillary analyses to investigate whether the findings reflected differential effects of FPz vs. Cz spindles instead of slow vs. fast spindles. Neither the overall density of FPz spindles, nor the overall density of Cz spindles, were associated with either overall speed or accuracy or their overnight enhancement (0.16 < *P* < 0.76). To further explore the relevance of spindle frequency, we ran ancillary analyses on the predictive value of the mean frequencies at FPz and at Cz for motor skill speed and accuracy. Children show a higher overall speed if they have a higher mean frequency of their spindles measures either at FPz (3.99 ± 1.95 correct sequences/Hz, *Z* = 2.046, *P* = 0.04) or at Cz (3.62 ± 1.60 correct sequences/Hz, *Z* = 2.268, *P* = 0.02). The mean spindle frequencies were not associated with overall accuracy (*P* = 0.48 and *P* = 0.10 for FPz and Cz respectively), nor with overnight enhancement of speed or accuracy (0.38 < *P* < 0.91).

In summary, overall performance is best in children with a high density of fast spindles and a low density of slow spindles. Children with a high density of slow spindles profit most from sleep to attain a higher accuracy.

### Slow wave characteristics and their association with performance baseline and overnight enhancement

Table 1 summarizes the characteristics of slow waves detected in S2, S3 and S4. Because of the frequency-specific associations of spindles with overall performance and sleep-dependent enhancement, it was of particular interest to investigate whether a similar frequency-specific effect of slow waves was present, i.e., whether their duration (inverse of frequency) mattered for performance. Indeed, significant effects were found only for slow wave duration.

With respect to overall performance (i.e., including all trials, not specific for overnight enhancement), children with a longer average duration of their slow waves had a lower overall speed, no matter whether the slow wave duration was derived from FPz (−0.102 ± 0.041 less correct sequences/ms longer duration, *Z* = −2.457, *P* = 0.014) or Cz (−0.084 ± 0.043 less correct sequences/ms longer duration, *Z* = −1.960, *P* < 0.050). Likewise, children with a longer average duration of their slow waves had a lower overall accuracy, significantly so for slow wave duration derived at FPz (−0.77 ± 0.30 lower % accuracy per milliseconds longer duration, *Z* = −2.567, *P* = 0.010) and almost significant for slow wave duration derived at Cz (−0.57 ± 0.30 lower % accuracy per milliseconds longer duration, *Z* = −1.900, *P* = 0.057). Given that the range of individual differences in the average duration of slow waves (FPz: 763–828; Cz: 752–822) covers up to 70 ms, the findings suggest slow wave duration-associated individual differences in speed of up to about six correct sequences and in accuracy of up to about 50%.

With respect to the overnight enhancement of performance, children with a longer average duration of their slow waves showed a stronger overnight increase in accuracy, significantly so for slow wave duration at Cz (0.36 ± 0.16% stronger increase in accuracy per milliseconds longer duration, *Z* = 2.25, *P* = 0.024) and almost significant for slow wave duration derived at FPz (0.32 ± 0.17% stronger increase in accuracy per milliseconds longer duration, *Z* = 1.882, *P* = 0.060). Slow wave duration was not associated with overnight changes in speed (FPz: *P* = 0.66; Cz: *P* = 0.42). Given the range of individual differences in the average duration of slow waves mentioned above, the findings suggest slow wave duration-associated individual differences in the overnight increase in accuracy of up to about 25%.

In summary, overall performance is best in children with a faster slow waves. Children with slower slow waves profit most from sleep to attain a higher accuracy.

### Association between individual differences in fast and slow spindle density with average slow wave duration

Given the findings overall performance is best in children with faster slow waves, a high density of fast spindles and a low density of slow spindles, *post hoc* correlations were calculated over the individual’s pairs of these slow wave and parameters. The average duration of slow waves measured at FPz was negatively correlated with the density of fast spindles (*r* = −0.40, *p* = 0.03) and almost significantly positively correlated with the density of slow spindles (*r* = 0.37, *p* = 0.05). The average duration of slow waves measured at Cz showed no significant correlation with the density of either fast spindles (*r* = −0.05, *p* = 0.80) or slow spindles (*r* = 0.10, *p* = 0.61). In summary, there is a significant association between the dominant frontal frequency of two characteristic sleep microstructural events with relevance for motor skill performance: the average duration of a slow waves measured and the density of fast spindles.

## Discussion

The present study set out to investigate the following questions. We hypothesized that motor skill enhancement is dependent on sleep in school-aged children. We moreover hypothesized that initial motor skill performance, and its enhancement after an interval without training, depend on the parameters that quantify the sleep-EEG macrostructure and microstructural properties of spindles and slow waves. Finally, to complement associational findings, we aimed to evaluate whether the hypotheses would be supported by an intervention aimed at manipulation of spindles and slow waves.

Similar to findings in adults (Walker et al., 2002; Van Der Werf et al., 2009b), the current report demonstrated children express offline enhancements in motor skill accuracy only if this interval includes a period of sleep. However, unlike previously reported in adults, children enhance their speed no matter whether the interval includes a period of sleep. In contrast to previous reports with similar results (Fischer et al., 2007; Wilhelm et al., 2008; Prehn-Kristensen et al., 2009), we do not interpret these results to indicate that children fail to show a speed enhancement over a period of sleep. Children do in fact show an enhancement of speed over a period of sleep, but as well over a period without sleep. Our interpretation is rather that children, like adults, do have the ability to enhance motor speed over a period of sleep, but the offline improvement can also be achieved across the different brain state of wakefulness (and thus perhaps by a different brain-state mechanisms). A speculative suggestion from our findings, that could be addressed in long-term follow-up studies on the development from childhood to adulthood, is that the capacity to improve performance without the necessity of sleep may be lost in adulthood. This suggestion is in line with recent findings indicating that procedural memory stabilizes during waking much faster in children than in adults (Ashtamker and Karni, 2013; Adi-Japha et al., 2014). Although children enhance their motor speed over periods of sleep and wake alike, sleep is required for an increase in accuracy (Figure 3).

An important new finding of the present study concerns the question of whether initial motor skill performance, or its enhancement after an interval without training, depend on specific aspects of the sleep-EEG microstructure. The results consistently indicate that individual differences in the dominant frequency of thalamo-cortical oscillations marks differences in both initial performance and sleep-dependent skill enhancement. Children with lower dominant frequencies of spindles and slow waves performed worse, as consistently indicated by the findings that children performed better if they had less slow spindles, more fast spindles and faster slow waves. The negative association between overall performance and the density of slow spindles is in line with a recent study by Kurdziel et al. (2013) who found, in 4-year children, that spindle density during a nap correlated negatively (*r* = −0.67) with baseline performance on a hippocampal-dependent visuospatial task resembling the card-deck “Memory” game. The hippocampus has also been implicated in sleep-dependent consolidation of motor sequence learning (Albouy et al., 2013b,c,d).

On average, characteristic oscillations in the EEG are slower in children than in adults and indeed also the peak frequency of sleep spindles increases as children mature (De Gennaro and Ferrara, 2003; Jenni and Carskadon, 2004; Tarokh and Carskadon, 2010). Our findings therefore suggest that dominant physiological frequencies of the characteristic sleep events may reflect trait-like markers of maturity within neuronal networks involved in cognition, including that associated with offline motor skill enhancement. It appears timely to consider large-scale multivariate follow-up studies to disentangle individual traits from developmental aspects, as well as common vs. differential involvement of spindle characteristics in motor skills, explicit memory and intellectual abilities (Geiger et al., 2011, 2012; Chatburn et al., 2013; Gruber et al., 2013; Hoedlmoser et al., 2014).

With respect to the overnight increase in performance, there appears to be a discrepancy at first sight between findings based on the density of slow and fast spindles vs. the findings based on the mean frequency of spindles. A stronger overnight increase in accuracy was associated with a higher density of slow spindles but not with a lower mean frequency of spindles. We interpret this finding as support for distinct types of spindles, as suggested by a bimodal distribution (Figure 5). The mean frequency depends on the number of both slow and fast spindles, and can be low irrespective of overall density. Overnight accuracy enhancement appears specifically associated with the abundance of slow spindles. The finding that the density of slow spindles, rather than fast spindles as in adults, is associated with the overnight increase in accuracy is interesting, since in children and adolescents, there is a slower frequency peak in the spindle-related sigma power (Jenni et al., 2005; Kurth et al., 2010). Thus, it may be that this leftward shift in the dominant spindle frequency curve, relative to adults, is involved in this differential association, and could still reflect similar overlapping consolidation mechanisms. Indeed, sleep spindle frequency in human adults has been associated with structural gray matter properties of the hippocampus. Moreover, surface EEG recorded spindles in human adults are associated with coinciding hippocampal activation. Should similar spindle-hippocampal associations be identified in child, this may provide one potential neural pathway through which spindle-related motor skill improvements are transacted in child, especially since the hippocampus is importantly involved in explicit motor skill learning (Walker et al., 2005; Steele and Penhune, 2010; Saletin et al., 2013).

Heib et al. (2013) showed a positive correlation between individual differences in the duration of the positive half-wave of the slow oscillation and their overnight changes in memory for word pairs. They speculated that a prolonged depolarizing up-state extends the time window for neuronal replay and thus enhances overnight memory improvement. No increase in the duration of slow oscillations in response to learning was found in this study, nor in a previous similar study (Mölle et al., 2009). These studies did not investigate whether individuals with longer positive half-waves might have had lower initial, pre-sleep, performance, and thus more room for overnight improvement similar to the current findings in children. Our present findings suggest that it may be important to investigate whether associations of sleep parameters with overnight improvements are secondary to associations of the same sleep parameters with initial performance. In the present study, the use of mixed effect multiple regression models allowed for a separation of these different associations.

Interestingly, the enhancement of accuracy over a period of sleep and of speed over a period of either sleep or wakefulness, is of a greater magnitude than has previously been reported in adults. The overnight improvement of speed, irrespective of sleep, was about 45%, which is more than twice the sleep-dependent speed improvement reported in the original study in adults (Walker et al., 2002). The overnight improvement in accuracy was 49%. Whereas no sleep-dependent change in accuracy reported in the original study in adults (Walker et al., 2002), later studies found accuracy improvements of up to 48% (Kuriyama et al., 2004). A parsimonious explanation of the findings is that participants that show an initial low performance, as is the case in the present study in children, have more headroom for improvement. This interpretation is supported by the fact that the strongest sleep-dependent increase in accuracy occurred in those that initially performed worst, i.e., those with lower dominant frequencies of spindles and slow waves. A recent study in 4-year old children also observed an inverse association between initial performance and sleep-dependent improvement (Kurdziel et al., 2013). As was the case for slow spindles (typical of young children) in our present study, they observed that sleep spindle density was negatively correlated with baseline performance and positively correlated with the change in memory performance across the nap period. In that study, children with a higher sleep spindle density initially performed worse and benefitted more from sleep for subsequent performance. Importantly, if associations of spindle and slow wave characteristics with initial performance are not accounted for, they may confound interpretation of their involvement in overnight enhancement.

The current study result need to be appreciated within the context of several inherent limitations. First, the sleep of children was so resistant to acoustic manipulation that we did not succeed in our aim to take the level of evidence for a role of spindles and slow waves in overnight a step further, from observational data to experimental intervention. The present findings confirm previous findings (Busby et al., 1994) suggesting that children have a much more powerful thalamic gate to shut off environmental monitoring during sleep.

A second limitation is that during the night of polysomnographic recording the children performed the task later in the evening than their habitual bedtime and slept relatively short. With respect to the late assessment, Figure 3 shows no systematically worse performance. The speed during both learning and recall in the evening did not differ from the speed during learning and recall in the morning, and the accuracy during learning in the evening did not differ from the accuracy during learning in the morning. These considerations support the interpretation that the lack of accuracy improvement in the morning-to-evening condition is specifically due to a lack of sleep. With respect to sleep duration, a recent systematic review on normal sleep patterns in children concluded that 11-year olds on average sleep 9 a night (Galland et al., 2012). Sleep duration was somewhat restricted in the present protocol due to the task assessment protocol with strict 12 h and 24 h intervals, so that the evening task assessment started at 10:00 PM. This resulted in a late bedtime as compared to their habitual bedtime (8:46 PM ± 00:21 min). Sleep duration may moreover have been somewhat restricted due to the excitement of the children about participating in a study that included sleeping a night in a Science Museum. The distribution of sleep stage durations in the present study was however very similar to those reported in previous studies on sleep in children (Fischer et al., 2007; Backhaus et al., 2008; Wilhelm et al., 2008). Ideally, a replication study would assess whether the reported associations hold if children are recorded at home according to their habitual sleep schedule.

A third limitation is that sleep was recorded in a non-shielded environment, which may have induced a larger number of epochs containing artifacts than would be expected in the environment of a well-controlled sleep-laboratory. A further limitation is that no extensive clinical evaluation on sleep disturbances was performed.

Finally, it should be noted that performing a motor skill task prior to bedtime may in itself alter the distribution of sleep spindles. Studies in humans and animals have consistently shown spindle activity to increase following training on several tasks, including the motor sequence tapping task used in the present study (Nishida and Walker, 2007; Barakat et al., 2011). Barakat et al. (2011) studied how sleep was affected by pre-sleep training on the same finger-tapping task that was used in the present study. They found that, compared to training on a control task, the motor sequence tapping task increased the density of fast spindles, while the density of slow spindles did not change. Subjects with the strongest training-elicited increase in fast spindle density showed the strongest sleep-dependent speed enhancement. Slow spindle density was not related to the sleep-dependent enhancement. Accuracy was not investigated. The association may be specific to the type of motor skill, because data presented by Tamaki et al. (2008; Table 1) suggest a decrease rather than increase in the number of fast spindles after training a mirror tracing skill. Moreover, although we cannot exclude the possibility that the motor skill task performance prior to bedtime increased spindle activity, it should be noticed that the functional relevance of such increase may be limited to the cortical area that are most prominently activated by the task, an area below the C4 electrode (Nishida and Walker, 2007).

In summary, the present findings indicate that even without sleep, children have the ability to increase the speed of their motor skills without training, a capacity that seems to be lost in adulthood. Moreover, whereas the majority of previous studies focused on sleep-dependent consolidation and enhancement, the present findings underscore the importance of investigating the associations of slower vs. faster oscillating spindles and slow waves with initial performance (Bódizs et al., 2005; Schabus et al., 2008), and the necessity to investigate how overnight improvements may be limited by high initial performance and enhanced by low initial performance. Overall, the present findings suggest that slower frequency oscillations of the characteristic sleep events may mark a less mature neuronal networks involved in motor skills and slower learning curves. This finding can be seen as a warning for a likely confound: if associations of spindle and slow wave characteristics with initial performance are not accounted for, they may confound interpretation of their selective involvement in overnight enhancement.

## Conflict of interest statement

The authors declare that the research was conducted in the absence of any commercial or financial relationships that could be construed as a potential conflict of interest.
